# Enhancing prehospital analgesia – advantages and further indications of nalbuphin

**DOI:** 10.1186/s13049-024-01227-9

**Published:** 2024-06-12

**Authors:** Gerrit Jansen, Marvin Deslandes, Martin Deicke, Julia Johanna Grannemann, Annika Hoyer, Matthias Kalmbach, André Kobiella, Thomas Plappert, Bernd Strickmann, Jochen Hinkelbein

**Affiliations:** 1 University Department of Anesthesiology, Intensive Care Medicine and Emergency Medicine, Johannes Wesling Klinikum Minden, Ruhr University Bochum, Hans-Nolte-Straße 1, 32429 Minden, Germany; 2 County of Osnabrueck, Am Schölerberg 1, 49082 Osnabrueck, Germany; 3 Department of Anesthesiology and operative Intensive Care Medicine, Hospital of Osnabrueck, Am Finkenhügel 1, 49076 Osnabrueck, Germany; 4 Emergency Medical Services, City and District of Guetersloh, Herzebrocker Strasse 140, 33324 Guetersloh, Germany; 5 Department of Biostatistics and Medical Biometry, Medical School OWL, Bielefeld University, Universitätsstraße 25, 33615 Bielefeld, Germany; 6 Emergency Medical Services, City and District of Fulda, Otfrid-von-Weißenburg-Str. 3, 36043 Fulda, Germany; 7 Emergency Department, Klinikum Fulda, Pacelliallee 4, 36043 Fulda, Germany; 8 Emergency Medical Services of the Order of Malta, Region Hesse, Schmidtstrasse 67, 60326 Frankfurt/Main, Germany; 9 Emergency Medical Services; City and District of Guetersloh, Herzebrocker Strasse 140, 33324 Guetersloh, Germany

Dear Editor,

the authors thank Rohat et al. for their Letter to the Editor and the specific interest in our publication “Effectiveness and safety of prehospital analgesia with nalbuphine and paracetamol versus morphine by paramedics - an observational study” [1].

As Rohat et al. correctly mentionend, the aforementioned study - like all retrospective observational studies - exhibits biases that must be taken into account in its careful interpretation. In observational studies - in contrast to prospective randomised studies - the patient collectives are generally not completely structurally identical. Hence, the analysis was adjusted for possible confounders as well as for the influence of exposure (group membership).

Despite numerous efforts to improve preclinical analgesia concepts, the rate of preclinical oligoanalgesia - as Rohat et al. correctly point out - remains unsatisfactorily high, often due to fear of complications of analgesic therapy [2]. However, due to its favourable pharmacodynamics, rapid onset, sufficiently long duration of action and unique safety profile with a low rate of side effects, nalbuphine appears to be well suited to complement the preclinical armamentarium of analgesics [1, 3]. In addition to analgesia, the authors believe that further prehospital use of nalbuphine could include sedation in the treatment of acute dyspnoea while avoiding respiratory insufficiency, as well as the antagonisation of µ-opioid receptor-associated side effects in the context of diacetylmorphine intoxication with simultaneous ĸ-receptor-mediated analgesia or sedation.

The authors - like Rohat et al. - are convinced that further prospective studies are urgently required in order to characterise the patient collectives and pain intensities of those patients who particularly benefit from nalbuphine in comparison to other µ-agonists, as well as any complications that may occur during the course of treatment within the hospital. The START-A-mnemonic proposed by Rohat et al. could be very helpful in this setting.

## Data Availability

Not applicable.
